# Retraction Note: Evolutionary analysis of rainstorm momentum and non-stationary variating patterns in response to climatic changes across diverse terrains

**DOI:** 10.1038/s41598-026-45406-3

**Published:** 2026-03-23

**Authors:** Chien-Lin Huang, Nien-Sheng Hsu

**Affiliations:** Department of Civil Engineering, National Taiwan University, No. 1, Sec. 4, Roosevelt Road, Taipei, 10617 Taiwan

Retraction of: *Scientific Reports* 10.1038/s41598-024-53939-8, published online 16 February 2024

The Editors have retracted this Article.

After publication concerns were raised that the data included in the Article was not generated by the Authors and that the manuscript was submitted without the agreement from the data generators. Further investigation by the Editors found evidence of manipulation of the authorship during the peer review process. The Editors therefore no longer have confidence in the contents of this Article.

None of the Authors responded to the Editors’ correspondence about this retraction.

